# Prostaglandin D2 delays CD8+ T-cell responses and respiratory syncytial virus clearance in geriatric cotton rats

**DOI:** 10.1128/jvi.01863-24

**Published:** 2025-01-17

**Authors:** Jonathan L. Miller, Cameron Leedale, Danyue Kang, Jingtao Lilue, Olivia E. Harder, Stefan Niewiesk

**Affiliations:** 1 Department of Veterinary Biosciences, College of Veterinary Medicine, The Ohio State University 198563 , Columbus, Ohio, USA; 2 Oujiang Laboratory 660404 , Wenzhou, China; Universite Laval, Laval, Quebec, Canada

**Keywords:** cotton rat, respiratory syncytial virus, aging, prostaglandin D_2_

## Abstract

Respiratory syncytial virus (RSV) infection is associated with increased rates of severe disease, hospitalization, and death in elderly individuals. Clearance of RSV is frequently delayed within this demographic, contributing to the more severe disease course. Geriatric cotton rats mimic this prolonged clearance kinetic and serve as a useful animal model for studying age-associated immunological deficits during RSV infection. Treatment with the cyclooxygenase (COX) inhibitor ibuprofen restores RSV clearance, indicating that inflammation contributes to impaired clearance in geriatric cotton rats. Here, we further characterize a compromised immune response in geriatric cotton rats and identify an inflammatory pathway that contributes to this deficiency. Dendritic cell (DC) activation and migration to mediastinal lymph nodes are decreased during early infection in geriatric cotton rats, resulting in delayed generation of cytotoxic T cells and virus clearance. Prostaglandin D_2_ (PGD2), which reduces DC migration through the elevation of D-type prostanoid 1 receptor (DP1 receptor), is elevated in the airways of infected geriatric cotton rats. Reducing PGD2 production by inhibiting COX-2 or PGD2 synthase improves RSV clearance kinetics through DC activation and RSV-specific CD8+ T-cell responses in geriatric cotton rats, whereas activation of DP1 receptor through an agonist resulted in delayed viral clearance in adult cotton rats. These results indicate that PGD2 contributes to delayed antigen presentation and CD8+ T-cell responses to RSV in geriatric cotton rats. Inhibiting PGD2 generation or signaling may be a useful mechanism of therapeutic intervention in elderly individuals.

**IMPORTANCE:**

Elderly adults are at increased risk of severe disease resulting from infection with respiratory syncytial virus (RSV), characterized in part by delayed clearance (removal of the virus from airways). Understanding the immunological factors that lead to this delayed clearance may allow for the development of therapies to improve disease outcomes in elderly individuals infected with RSV and other respiratory viruses. Here, we describe an inflammatory pathway in geriatric cotton rats, the preferred small animal laboratory model for RSV, that impairs the generation of an effective immune response. We show that inhibiting this inflammatory pathway in geriatric cotton rats improves immune parameters and speeds clearance of RSV. These results contribute to our understanding of delayed RSV clearance in elderly individuals with possible applications for improving immune responses to RSV in clinical settings.

## INTRODUCTION

Respiratory syncytial virus (RSV) is one of the leading causes of acute respiratory infection in elderly people. Immunity following RSV infection is short-lived, leading to recurrent infections over a lifetime. In adults over 65 years of age, these infections are associated with an increased risk of severe clinical disease and mortality rates. Although clinical presentation in most infected adults is limited to mild upper respiratory tract disease, severe infection in the elderly can progress to bronchiolitis and pneumonia. Hospitalization of severely affected elderly individuals can be associated with substantial costs to individuals and/or the healthcare system, frequently requiring oxygen supplementation or ventilatory support. In the United States, RSV causes an estimated 6,000–10,000 deaths and 60,000–160,000 hospitalizations annually among the elderly with an even higher rate for outpatient visits (1–4). Increased severity of respiratory infections has been tied to several age-related declines in innate and adaptive immune responses (5, 6), but the mechanism of impaired responses to RSV remains unclear.

The cotton rat (*Sigmodon hispidus*) is the preferred small animal model for studying RSV infection. The similarities to humans in terms of infection and immune response have made cotton rats an important animal model in studying the efficacy of vaccines and prophylactic treatments. The predictive quality of the model has obviated the need for safety and efficacy studies in nonhuman primates in some cases (7–10). As in humans (but unlike mice), cotton rat bronchiolar epithelium is composed of ciliated columnar cells that are the target of RSV infection in both the nose and the lung (11, 12). Reinfection is also a feature of RSV in cotton rats, with complete protection in the upper airway declining after 8 months (13). Cotton rats are approximately 100-fold more permissive to RSV infections than BALB/C mice (14). As in humans, neutralizing antibodies protect against secondary infection but CD8+ T cells are critical for RSV clearance. During primary infection of cotton rats, depletion of CD4+ T cells does not affect virus replication, whereas the depletion of CD8+ T cells severely delays viral clearance (15). The major CD8+ T-cell epitopes are found on the fusion and nucleoprotein with minor epitopes being present on the M2 protein (16–18).

Consistent with our own findings, other laboratories have reported that RSV infection of geriatric cotton rats demonstrates key features seen in humans. In cotton rats 8 months of age and older, RSV clearance is delayed in both lung and nasal mucosal tissue (19–21). Laboratory-housed cotton rats have a life expectancy of 9–14 months, with an average in our laboratory of 11 months (22). There have been limited studies investigating the effects of aging on the immune response to RSV in the cotton rat. Kinetics of expression of several cytokines is reported to be shifted in geriatric compared with adult cotton rats, including elevated GRO (CXCL1) expression and delayed peak expression of IFN-γ, IL-6, and IL-10 (21). It has also been determined that immunization efficacy declines in geriatric cotton rats (19). Both CD4+ and CD8+ T-cell percentages were found to be lower in geriatric cotton rats in lymphoid tissue compared with adults (23). However, CD8+ T-cell responses to RSV have not been evaluated in geriatric cotton rats.

Studies with aged mice indicate one age-related mechanism for how cytotoxic T-cell responses can be affected during respiratory viral infections. In one study, C57BL/6 mice infected with SARS CoV-1 or influenza A virus exhibited a decreased virus-specific CD8+ T-cell response in the lungs of aged mice (24). Investigators in that study identified an age-dependent elevation of pulmonary prostaglandin D_2_ (PGD2) as a contributing factor to the impaired CD8+ T-cell responses by interfering with lung dendritic cell (DC) migration to regional lymph nodes. PGD2 inhibition of DC CCR7 expression and chemotaxis to secondary lymphoid tissue has been characterized in several tissues, including skin and airways, mediated through the activation of D-type prostanoid receptor 1 (DP1) (25–27).

Our laboratory has previously shown that oral ibuprofen treatment improves RSV clearance in geriatric cotton rats, allowing them to clear the virus from lung and nasal samples within 6 days as is seen in adult animals (20). Ibuprofen, a pan-cyclooxygenase (COX, alternatively known as prostaglandin-endoperoxide synthase) inhibitor, interferes with the generation of prostaglandin H2 (PGH2) from arachidonic acid. Production of downstream metabolites (eicosanoids, including PGD2) from PGH2 is decreased as a consequence of ibuprofen treatment. Depletion of CD8+ T cells in geriatric cotton rats abolished any beneficial effect of ibuprofen, providing indirect evidence that the therapeutic effect COX inhibition results in improved cytotoxic T-cell responses.

We hypothesized that PGD2 slows the generation of CD8+ T-cell responses in geriatric cotton rats by inhibiting DC activation and migration. In this study, we quantified RSV-specific CD8+ T-cell responses and DC activation in adult and geriatric cotton rats. We then investigated the role of PGD2 in impaired immune responses to RSV in geriatric cotton rats.

## MATERIALS AND METHODS

### Animals

Inbred cotton rats (*Sigmodon hispidus*) were purchased from Inotiv (formerly Envigo; West Lafayette, IN) and maintained in a University Laboratory Animal Resources barrier facility at Ohio State University. Cotton rats were housed in NexGen Rat 900 polysulfone microisolator cages (Allentown Inc., Allentown, NJ) with 12:12 hour light cycles, 22°C ± 2°C, and 20%–70% relative humidity. Age ranges were 1–4 months for adult animals and >8 months for geriatric animals. Both sexes were used in all experimental groups.

### Chemicals

NS-398 (COX-2 antagonist; Cayman Chemicals, Ann Arbor, MI), TFC 007 (H-PGDS inhibitor; Tocris Bioscience, Bio-Techne, Minneapolis, MN), and BW245C (DP1 receptor agonist; Cayman) were reconstituted per manufacturer instructions. All drugs were administered intraperitoneally on the indicated days with a dosing interval of 24 hours at dosages of 5 mg/kg for NS-398, 20 mg/kg for TFC 007, or 1 mg/kg for BW245C.

### Virus and infection

Stocks of RSV-A2 (NCBI taxonomy ID: 11259) were propagated in HEp-2 cells in minimum essential medium (MEM; Thermo Fisher Scientific, Rockford, IL) with 2% fetal bovine serum and stored in liquid nitrogen as described previously (15). Cotton rats were anesthetized via isoflurane inhalation and inoculated intranasally with 10^5^ TCID_50_ RSV-A2 diluted in PBS for an inoculation volume of 100 µL.

### Tissue culture infectious dose 50 (TCID_50_) assay

Titers of RSV stocks, or homogenates of left lung or nasal turbinates, were determined by standard TCID_50_ assay. Briefly, 10-fold serial dilutions of viral stocks or homogenized tissues in 500 µL MEM were plated on 48-well plates of 80% confluent HEp-2 cells, using six wells per concentration. After incubating for 1 hour at 37°C and 5% CO_2_, the cells were washed three times with PBS/0.1% FCS, and 500 µL MEM/2% FCS was added per well. Plates were incubated at 37°C and 5% CO_2_, and the media was replaced on day 3. Plates were scored for cytopathic effect by light microscopic examination on day 5. TCID_50_ was calculated using the Reed-Muench method (28).

### Preparation of cells from tissues

Cotton rats were euthanized via carbon dioxide inhalation followed by exsanguination. For experiments utilizing flow cytometry as the downstream application, the indicated tissues were collected, washed in PBS, and trimmed of adjacent adipose and connective tissues. All lung lobes were finely diced with a scalpel blade and digested in Hank’s balanced salt solution with 0.1 mg/mL DNase I (MilliporeSigma, Burlington, MA) and 1 mg/mL collagenase (MilliporeSigma) at 37°C for 45 minutes. Mediastinal lymph nodes (MLN), spleen, and digested lungs were pressed through 100 µm mesh cell strainers (Fisher Scientific) to generate single-cell suspensions. The cells were washed three times with PBS and enumerated by trypan blue exclusion and light microscopic examination.

For quantification of viral titer, the left lung lobe and nasal turbinate mucosa were collected, weighed, and homogenized in 2 mL of MEM. Lung was homogenized with a Precellys 24 tissue homogenizer (Bertin Technologies, France) maintained at 4°C with a Cryolys® Evolution (Bertin) cryogenic cooling system and using parameters recommended by the manufacturer. Nasal turbinate mucosa samples were hand-homogenized using a CoorsTek mortar and pestle (Golden, CO) on ice with sterile sand. RSV titers were determined by TCID_50_ assay on HEp-2 cells.

### Antibodies and flow cytometry

CD8+ T cells were characterized immunophenotypically by flow cytometry as CD8+ (monoclonal mouse IgG2a anti-cotton rat CD8α, clone JG12, R&D Systems, Minneapolis, MN) and CD4- (monoclonal mouse IgG2b anti-cotton rat CD4, clone 695542, R&D Systems) T cells. DCs were characterized immunophenotypically as MHC II+ (cross-reactive monoclonal mouse IgG2a anti-mouse/rat MHC II (I-Ek), clone 14-4-4S, Thermo Fisher), CD11c+ (cross-reactive polyclonal rabbit IgG anti-human/mouse/rat CD11c, immunogen amino acids 146–342 of ITGAX, Thermo Fisher), and Siglec F- (cross-reactive polyclonal rabbit IgG anti-human/mouse Siglec F, immunogen amino acids 8–39 of human SIGLEC5, Thermo Fisher). Proportion of lung DCs expressing CCR7 (cross-reactive polyclonal rabbit IgG anti-human/mouse/rat CCR7, immunogen amino acids 25–59 of mouse CCR7, Thermo Fisher) were measured within the MHC II+/CD11c+/Siglec F- population. Percentages of CD8+ T cells expressing IFN-γ (polyclonal goat IgG anti-cotton rat IFN-γ, immunogen amino acids 21–170 of cotton rat IFN-γ, R&D Systems) were measured in RSV peptide-stimulated and unstimulated cell suspensions (see intracellular cytokine staining, below).

All staining steps were performed at 4°C. In total, 1 * 10^6^ cells were washed twice with PBS/0.1% FCS followed by surface staining for 30 minutes with the indicated antibodies. All flow cytometry experiments were performed on an Attune NxT Flow Cytometer (Thermo Fisher). A minimum of 10,000 events were analyzed for each tissue. Analysis of measurements was conducted with Attune Cytometric Software.

### Peptides and intracellular cytokine staining

RSV-specific peptides were synthesized and purchased from Pierce Custom Peptides (Thermo Fisher). Peptide sequences used for immunodominant F protein epitopes were F protein amino acid 247–261 (VSTYMLTNSELLSLI) and F protein amino acid 253–265 (TNSELLSLINDMP) and those for immunodominant NP epitopes were NP protein amino acid 306–314 (NPKASLLSL) and NP protein amino acid 360–368 (ENGVINYSVLD) (16, 17).

For measurement of IFN-γ production in CD8+ T cells, 1 * 10^6^ cells were plated per well of a 96-well plate in 200 µL RPMI with 2% normal cotton rat serum and 20 µg/mL Brefeldin A (Invitrogen, Carlsbad, CA). Single-cell suspensions from each tissue were co-incubated with 5 µg/mL of each peptide and or left unstimulated. Following overnight incubation at 37°C, the cells were collected, washed twice with PBS/0.1% FCS, and surface stained for CD8 and CD4. Cells were then washed and fixed with a Cytofix/Cytoperm fixation/permeabilization kit (BD Biosciences, Franklin Lakes, NJ) following the manufacturer’s instructions. Permeabilized cells were stained for IFN-γ at 4°C for 30 minutes.

### Quantification of gene expression

The left lung lobe was collected from cotton rats at the indicated time point post-RSV infection and placed in 2 mL RNAprotect (QIAGEN, Hilden, Germany) at room temperature. For tissue homogenization, 15–20 µg of each lung sample was removed using sterile dissection instruments and placed in 2 mL Precellys® bead tubes (Bertin) with 600 µL Buffer RLT plus (QIAGEN) and 6 µL 2-mercaptoethanol and homogenized with a Precellys 24 tissue homogenizer (Bertin) at room temperature, followed by passage of the homogenate through QIAshredder tubes (QIAGEN). RNA was isolated using RNeasy Plus Mini extraction kits (QIAGEN) and following manufacturer instructions, with a final elution volume of 30 µL. RNA concentrations were measured using a NanoDrop One spectrophotometer (Thermo Fisher). First-strand complementary DNA (cDNA) was generated from 1 µg of each lung RNA sample using iScript cDNA Synthesis kits (Bio-Rad, Hercules, CA) following manufacturer-recommended instructions and thermal cycling conditions. cDNA concentrations were measured using a NanoDrop One spectrophotometer (Thermo Fisher).

Absolute quantification of the expression of interleukin-6 (IL-6), COX-2, and hematopoietic prostaglandin D_2_ synthase (hPGDS) was measured from lung cDNA using digital droplet polymerase chain reaction (ddPCR). Reactions were prepared using 300 ng of cDNA and ddPCR Multiplex Supermix (Bio-Rad). All primer and probe sets were ordered from Integrated DNA Technologies (Coralville, IA). For all reactions, primer concentrations were 900 nM and probe concentrations were 250 nM. For cotton rat IL-6 (GenBank accession AF421389), the forward primer sequence was ACACTTAGGCACAGCATACTA, the reverse primer sequence was GAGGACCAAGACCATCCAAC, and the probe sequence was TGCCGAGTAGACCTCATGGTGATC. For cotton rat COX-2 (GenBank accession AY065644.1), the forward primer sequence was TTGGAGCACCATTCTCCTTG, the reverse primer sequence was ATCTCAAAGCCCACTTGTCC, and the probe sequence was CCTCAGTACTGGAAGCCAAGCACT. For cotton rat hPGDS (GenBank accession PQ431520), the forward primer sequence was AGCCTGACTTGTTGGACATC, the reverse primer sequence was TCACTGCGGAAGAAAGTCCAAGCT, and the probe sequence was GTTTGGTCTGAGGCCTCTTT. Droplets were generated with a QX200 droplet generator (Bio-Rad). PCR amplification was performed with a C1000 thermal cycler (Bio-Rad) using an enzyme activation step at 95°C for 10 minutes, 40 cycles with a denaturation temperature of 94°C for 30 seconds and an annealing and extension temperature of 65.6°C for 90 seconds, and an enzyme deactivation step at 98°C for 10 minutes. A QX600 droplet reader (Bio-Rad) was used to measure the proportion of fluorescent droplets for each gene fragment. The results were analyzed using QX Manager Standard Edition 2.0.0 (Bio-Rad) with thresholds for positive events set automatically by the software. The total event count for all samples was >10,000. The concentration of IL-6, COX-2, and hPGDS was calculated as copies/ng of cDNA.

### Bronchoalveolar lavage PGD2 immunoassay

Cotton rats were euthanized at the indicated time points. The trachea was transected in the mid-cervical region and cannulated. The lungs were lavaged with 1 mL PBS. PGD2 was quantified in these bronchoalveolar lavage (BAL) samples using an enzyme-linked immunosorbent assay (ELISA) following the manufacturer’s instructions (Cayman).

### Statistical analysis

Data for all groups of cotton rats in all figures are expressed as the mean ± standard deviation. Statistical analyses were performed using GraphPad Prism 10 (GraphPad Software, La Jolla, CA). Differences in mean values between groups were analyzed with a Student’s *t* test. For experiments comparing >2 independent groups, a one-way analysis of variance was used, followed by Tukey’s multiple comparison post-hoc test. *P* values of > 0.05 were considered statistically significant.

## RESULTS

### RSV-specific CD8+ T-cell responses are delayed in geriatric cotton rats

**Fig 1:**
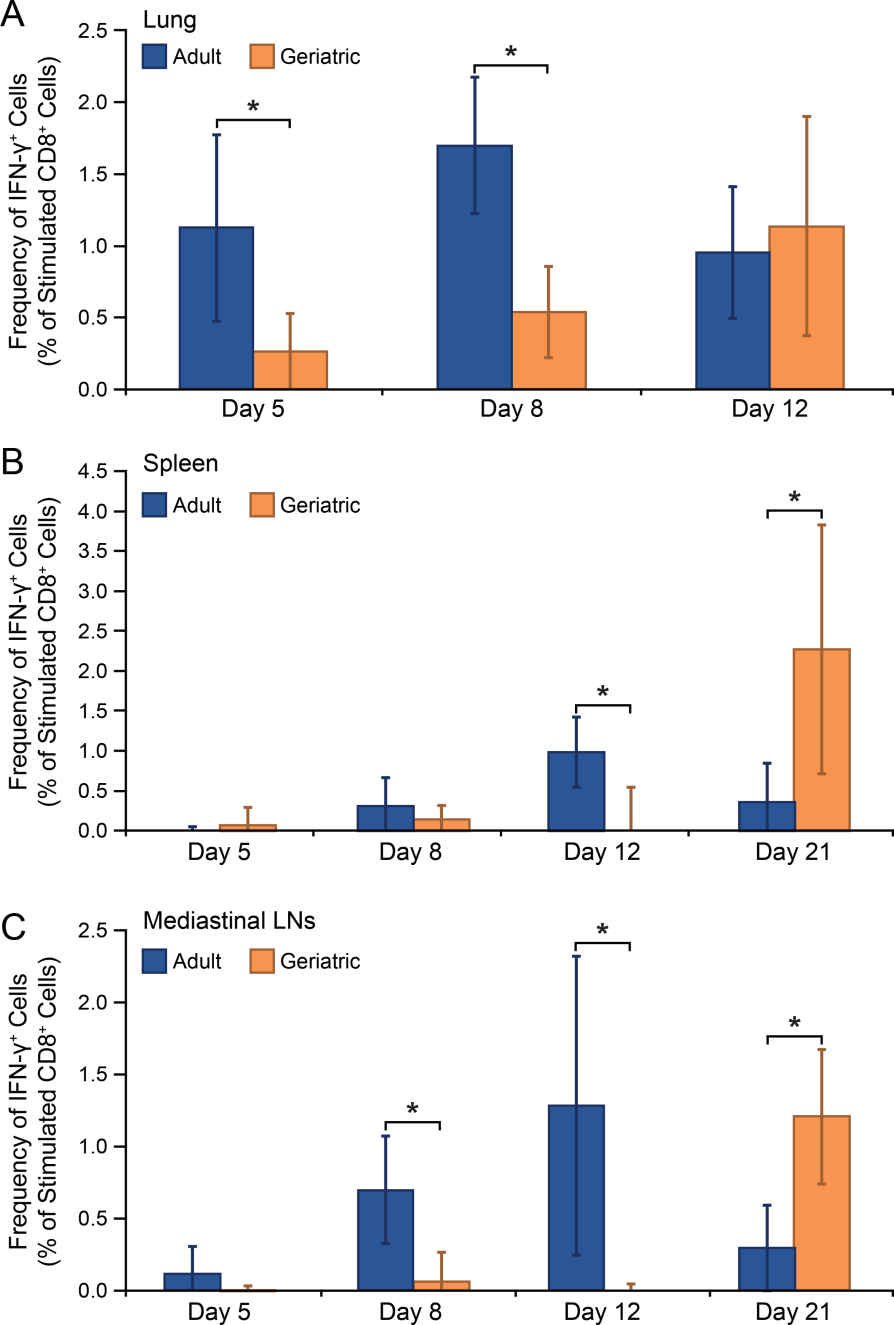
RSV-specific CD8+ T-cell responses in the lungs, spleen, and MLN are delayed in geriatric cotton rats. At the indicated days post-RSV infection, cotton rats were euthanized, and the tissues were collected. Single-cell suspensions were plated overnight with Brefeldin A and in the presence (stimulated) or absence (unstimulated) of immunodominant MHC-I-restricted peptides from the RSV F and NP proteins. Flow cytometry was performed measuring the percentage of CD8+ T cells expressing IFN-γ. The percentage of CD8+ T cells expressing IFN-γ from matching unstimulated samples (indicative of background/nonspecific labeling) was subtracted from stimulated samples. RSV-specific CD8+ T cells were first detectable in lung of adult animals, followed by appreciable populations in the mediastinal lymph nodes and spleen. RSV-specific CD8+ T cell percentages in geriatric animals were significantly lower (*P < 0.01) until after day 8 post-infection in lung, and until after day 12 post-infection in MLN and spleen. (*n* = 3 for geriatric day 21 in B and C. *n* = 5 for all other data points.)

Clearance of RSV from lung and nasal turbinate is delayed in geriatric cotton rats. Treatment with the pan-cyclooxygenase (COX) inhibitor ibuprofen eliminates this delay, implicating an age-associated elevated inflammatory state (inflammaging) as a contributor to impaired RSV clearance (20). Depletion of CD8+ T cells in geriatric cotton rats eliminated the restorative effect of ibuprofen on RSV clearance, indirectly suggesting that ibuprofen’s therapeutic effect relates to improving CD8+ T-cell responses. To directly investigate whether CD8+ T-cell responses are impaired in geriatric cotton rats, we measured CD8+ T lymphocytes in lung, MLN, and spleen at various days post-infection in adult and geriatric animals. Single-cell suspensions of tissues were plated overnight in the presence of immunodominant MHC-I-restricted RSV peptides. Flow cytometry was performed, gating for CD8+ T cells and measuring the proportion that expressed IFN-γ as a marker of activation. In all tissues, development of CD8+ T-cell responses against these RSV peptides was delayed (Fig. 1). Adult cotton rats exhibited a robust RSV-specific CD8+ T-cell response in lung by day 5 post-infection, MLN (the most proximal tertiary lymphoid tissue) by day 8 post-infection, and spleen by day 12 post-infection. By contrast, CD8+ T-cell responses were significantly lower in the lung of geriatric animals until day 12 post-infection and were not detectable in lymphoid tissues until after day 12 post-infection.

### Lung dendritic cell activation and migration to mediastinal lymph nodes is decreased in geriatric RSV-infected cotton rats

**Fig 2:**
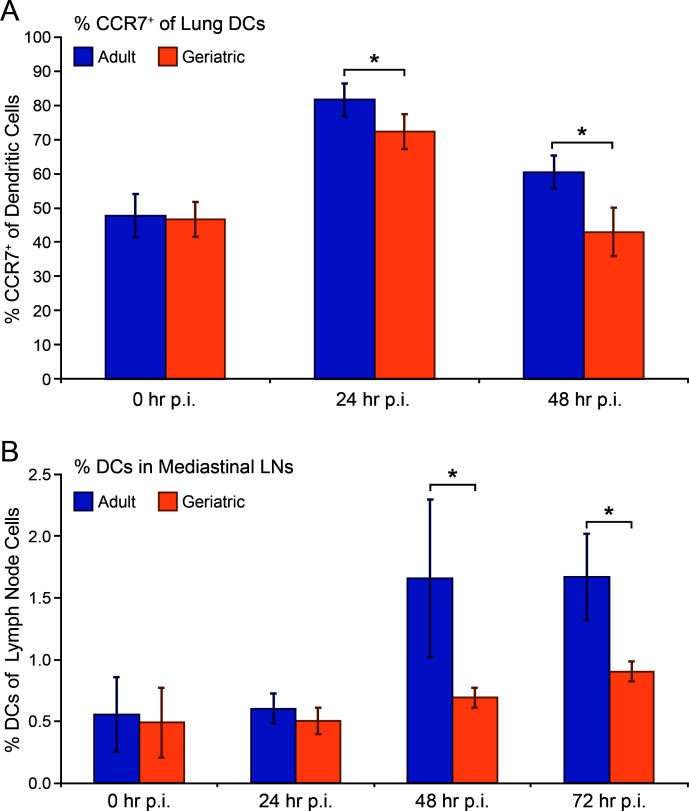
Lung DC activation and migration to lymph nodes are impaired in geriatric cotton rats. Tissues were collected at the indicated time points post-RSV infection, with 0 hours post-infection representing uninfected cotton rats. (**A**) A significantly lower (*P < 0.01) percentage of lung DCs (CD11c+/MHC-II+/Siglec F-) express CCR7, a marker of DC activation and migration to lymphoid tissues, at 24 and 48 hours post-infection in geriatric cotton rats compared with adults. (**B**) The proportions of DCs within MLN of adult cotton rats increase to significantly higher percentages (*P < 0.01) at 48 and 72 hours post-infection compared with geriatric animals, suggesting decreased DC migration from the lung in geriatric cotton rats. (*n* = 4 for adult 0 hours p.i. in A. *n* = 5 for all other data points.)

Antigen presentation and co-stimulation by activated DCs are necessary for the generation of a CD8+ T-cell response. We hypothesized that lung DC activation and migration to regional lymph nodes is impaired in RSV-infected geriatric cotton rats, contributing to the observed delay in CD8+ T-cell responses. To investigate lung DC activation, we measured CCR7 expression in lung DCs by flow cytometry. The chemokine receptor CCR7 is strongly upregulated in activated DCs and drives migration toward draining lymph nodes. At days 0, 1, and 2 post-infection with RSV, we collected lungs from adult and geriatric cotton rats. We generated single-cell suspensions and performed flow cytometry, measuring the percentage of CCR7+ cells among the DC population (CD11c+/MHC-II+/Siglec F-). Although DC activation was increased in both adult and geriatric cotton rats at 24 hours post-infection, it was significantly higher in adult animals. By 48 hours post-infection, the percentage of activated lung DCs in geriatric animals had returned to baseline levels but remained significantly higher in adults (Fig. 2A).

We measured DC populations in MLN to examine whether decreased DC activation resulted in delayed or decreased migration to draining lymph nodes. At days 0–3 post-infection with RSV, adult and geriatric cotton rats were euthanized, and MLN were collected. Flow cytometry was performed on single-cell suspensions, measuring the percentage of DCs (CD11c+/MHC-II+) of the total population. Adult cotton rats exhibited a robust increase in the DC proportion within MLN at 48 and 72 hours post-infection, and the percentage of DCs was significantly higher than geriatric animals at those time points (Fig. 2B). Collectively, these results indicate that pulmonary DC activation and migration of RSV-infected geriatric cotton rats are impaired, presumably driving delayed CD8+ T-cell responses.

### Elevated PGD2 in cotton rats contributes to delayed RSV clearance

**Fig 3:**
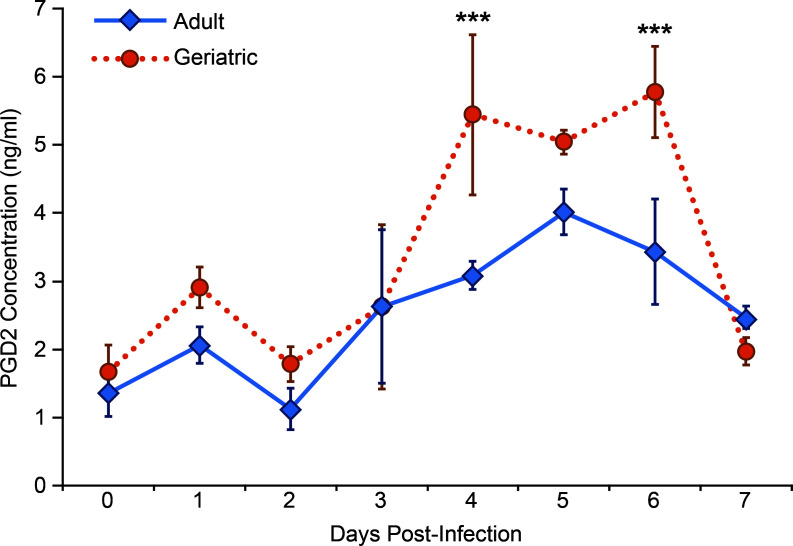
PGD2 is elevated in the airways of geriatric cotton rats. At the indicated time point post-RSV infection, adult and geriatric cotton rats were euthanized, and airways were sampled by BAL with 1 mL PBS. PGD2 concentrations were measured by ELISA. Day 0 values represent cotton rats administered PBS intranasally and euthanized at 2 days post-inoculation. PGD2 concentrations are significantly higher in geriatric cotton rats than in adults from days 4–6 post-infection (***P < 0.005). (*n* = 5 for all time points).

**Fig 4:**
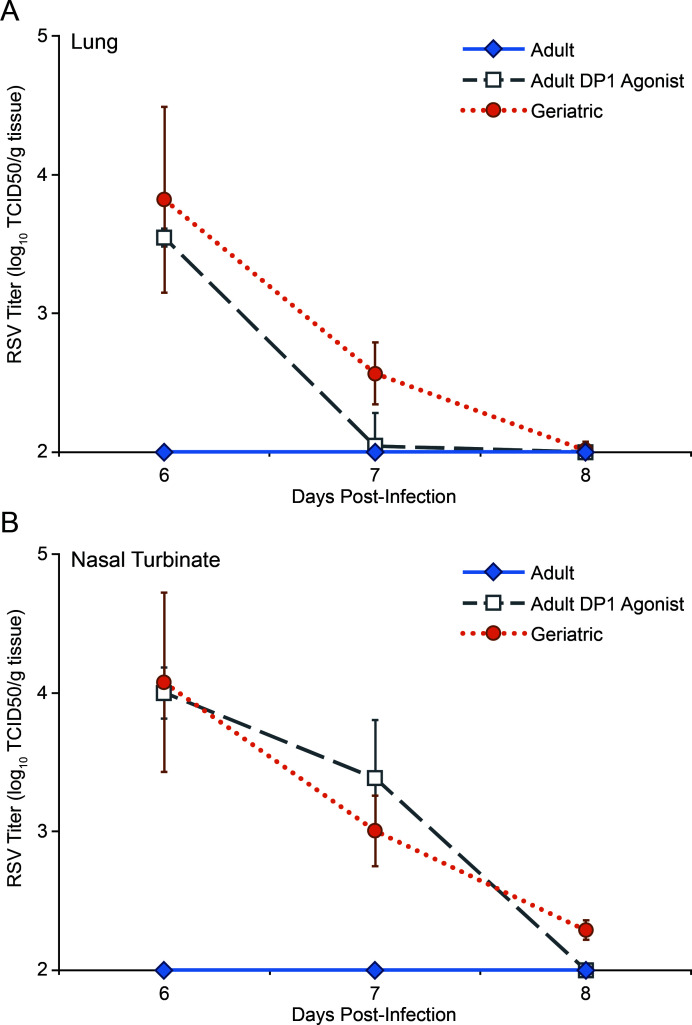
Prostaglandin D_2_ receptor 1 (DP1) agonism delays RSV clearance in adult cotton rats. The DP1 receptor agonist BW245C was administered intranasally to adult cotton rats daily from days 3–5 post-RSV infection. RSV titers were measured in lung (**A**) and nasal turbinates (**B**) by TCID_50_ assay at the indicated time points. Untreated adult cotton rats clear the virus below detectable thresholds by day 6 post-infection in both lung and nasal turbinate. Treatment with BW245C results in delayed clearance in both tissues, similar to geriatric cotton rats. (*n* = 5 for all adult untreated and DP1 agonist-treated time points. *n* = 3 for geriatric days 6 and 7. *n* = 4 for geriatric day 8.)

Because pan-COX inhibition results in improved RSV clearance kinetics in geriatric cotton rats, we hypothesized that age-associated elevations in an eicosanoid contribute to delayed clearance. Previous studies have demonstrated that increased PGD2 concentrations in aged mice delay the clearance of respiratory viruses by impairing DC migration and T lymphocyte activation (24, 29). This inhibitory effect of PGD2 on DCs, through activation of DP1, has been similarly demonstrated in other systems and conditions (25, 30). We measured PGD2 concentrations by ELISA in geriatric and adult cotton rats from days 0–7 post-infected with RSV. Bronchoalveolar lavage (BAL) samples from uninfected geriatric and adult cotton rats contained a similar concentration of PGD2. However, from days 4–6 post-infection with RSV, BAL samples from geriatric cotton rats contained a significantly higher concentration of PGD2 than adults (Fig. 3). To investigate the functional significance of elevated PGD2 in the airways of infected cotton rats, we administered a PGD2 analog, the DP1 agonist BW245C, to adult cotton rats. Naive adults were infected with RSV, and BW245C was administered intranasally from days 3–5 post-infection. We measured RSV titers in lung and nasal turbinate to determine viral clearance kinetics. Although untreated adult cotton rats cleared RSV from both tissues below detectable thresholds by day 6 post-infection, BW245C-treated adults still had detectable titers by day 7 post-infection, similar to geriatric cotton rats (Fig. 4).

### Inhibiting PGD2 production in geriatric cotton rats improves RSV clearance

**Fig 5:**
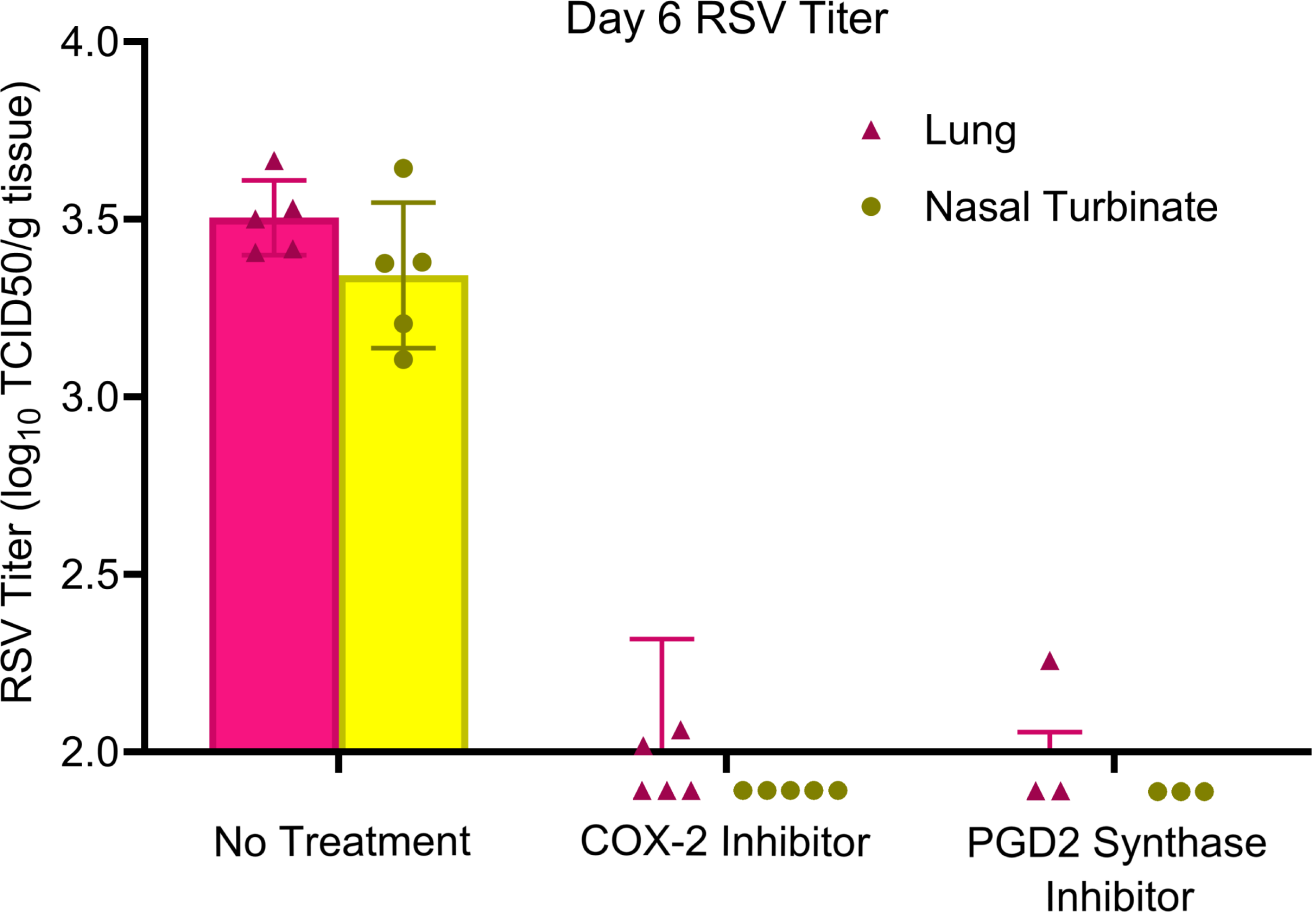
Inhibiting PGD2 production improves RSV clearance in geriatric cotton rats. From days 0–4 post-RSV infection, geriatric cotton rats were treated intraperitoneally with the COX-2 inhibitor NS-398, the PGDS inhibitor TFC 007, or PBS (no treatment control). At day 6 post-infection, RSV titers in lung and nasal turbinate were measured by TCID_50_ assay. Mean titers for groups treated with either the COX-2 inhibitor or PGDS inhibitor were below the sensitivity threshold of the assay, whereas titers in untreated geriatric cotton rats were still readily detectable. (*n* = 5 for No Treatment and COX-2 Inhibitor groups. *n* = 3 for PGD2 Synthase Inhibitor group. Data points displayed below the x axis represent animals with titers lower than the detectable threshold of the assay, with values of 0 used for mean and standard deviation calculation.)

Having shown that PGD2 is increased during RSV infections in geriatric cotton rats and that a DP1 agonist impairs RSV clearance, we next examined whether decreasing PGD2 concentrations in geriatric animals would restore viral clearance. We suppressed the generation of PGD2 by administering inhibitors of enzymes along its synthetic pathway. The COX enzymes are responsible for the conversion of arachidonic acid to PGH2, an intermediate precursor to PGD2. Given that PGD2 is elevated in airways of infected geriatric cotton rats but not in uninfected animals (Fig. 3), we presumed that the inducible isozyme COX-2 was more important than COX-1 in age-associated differences in the synthetic pathway. We treated RSV-infected geriatric cotton rats intraperitoneally with the COX-2 inhibitor NS-398 daily from days 0–4 post-infection. To determine if these treated geriatric cotton rats were able to clear RSV infection in the same timeframe as adults, we collected lung and nasal turbinates at day 6 post-infection and measured RSV titers. In both tissues, NS-398-treated geriatric cotton rats had cleared infection to below-detectable titers, whereas titers in untreated geriatric animals were still readily detectable (Fig. 5). We next wanted to confirm that PGD2, and not another eicosanoid derived from PGH2, is responsible for delayed RSV clearance in geriatric animals. PGH2 is converted to PGD2 by the prostaglandin D_2_ synthase (PGDS). We treated geriatric cotton rats intraperitoneally from days 0–4 post-RSV infection with the hematopoietic PGDS inhibitor TFC 007. At day 6 post-infection, RSV titers in lung and nasal turbinate were measured. Like COX inhibitor-treatment, PGDS inhibition resulted in the clearance of RSV infection by day 6 (Fig. 5), confirming more specifically that PGD2 is the primary eicosanoid responsible for delayed RSV clearance in geriatric cotton rats.

### COX-2 inhibition improves lung DC maturation and RSV-specific CD8+ T-cell responses

**Fig 6:**
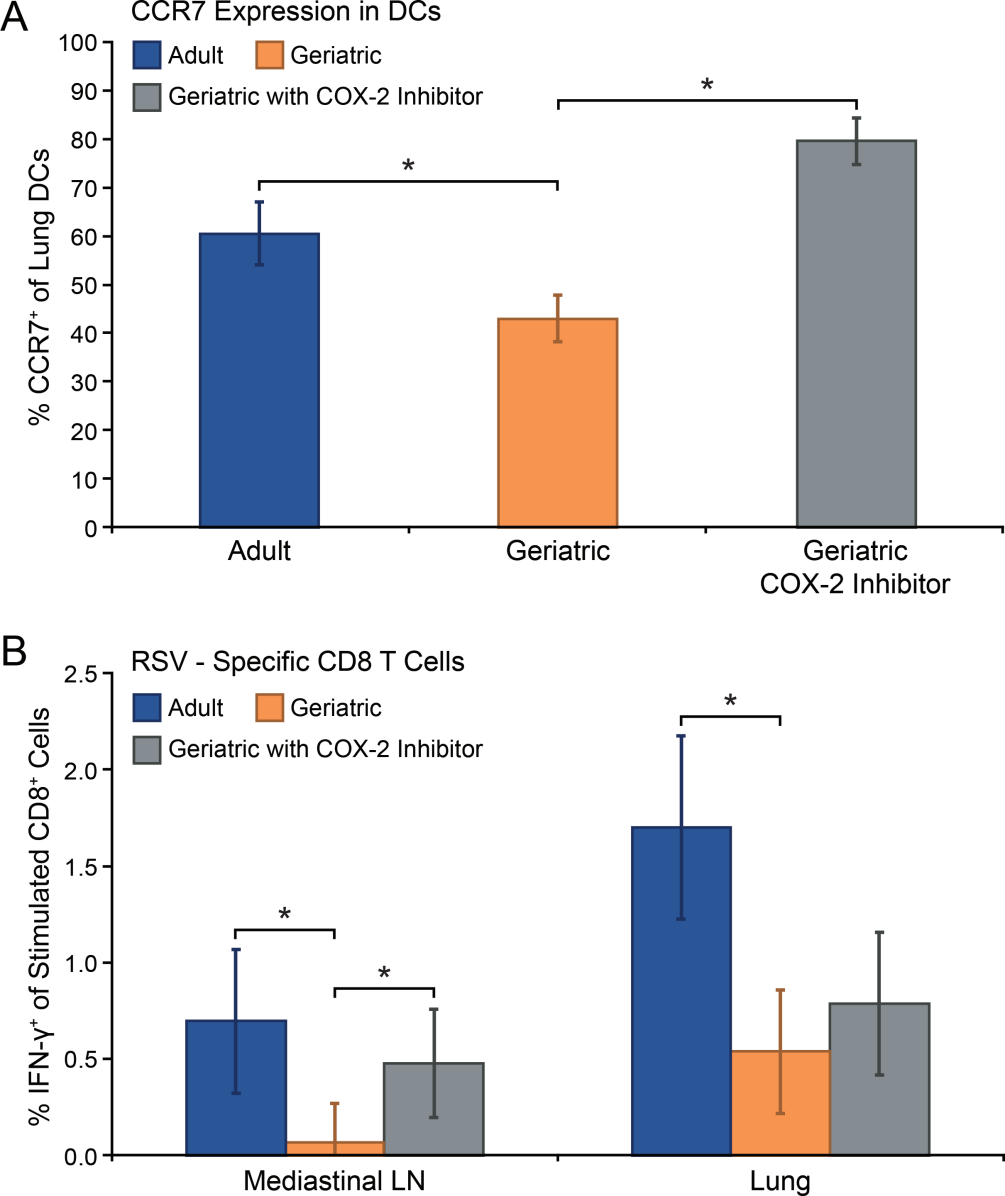
COX-2 inhibition increases pulmonary DC CCR7 expression and RSV-specific CD8+ T cell responses. RSV-infected geriatric cotton rats were treated daily with the COX-2 inhibitor NS-398 intraperitoneally from days 0–1 (**A**) or 0–4 (**B**). (A) At day 2 post-infection, a cohort was euthanized, and the lungs were collected to measure CCR7 expression in DCs (CD11c+/MHC-II+/Siglec F-) by flow cytometry. Compared with untreated geriatric cotton rats, NS-398 treatment resulted in higher CCR7-expressing lung DCs (*P < 0.01). (B) At day 8 post-infection, lung and MLN were collected to measure RSV-specific CD8 T-cell responses. The percentage of RSV-specific CD8+ T cells was calculated as the increase in the percentage of IFN-γ expressing CD8+ T cells in peptide-stimulated samples from unstimulated matching controls. COX-2 inhibition resulted in an increased RSV-specific CD8+ T-cell response in MLN (*P < 0.01) but not the lung. (*n* = 5 for all data points).

We next investigated the mechanism by which PGD2 impairs RSV clearance in geriatric cotton rats. DP1 activation by PGD2 results in impaired DC CCR7 expression and migration and has been shown to inhibit CD8+ T-cell responses in respiratory viral infections (24, 29). We treated geriatric cotton rats with NS-398 and measured lung DC CCR7 expression and RSV-specific CD8+ T-cell responses to confirm whether PGD2 was negatively affecting these metrics in our model. Compared with untreated geriatric cotton rats, those treated with NS-398 exhibited significantly higher CCR7 expression in lung DCs at 48 hr. post-RSV infection (Fig. 6A). NS-398-treated geriatric cotton rats also had a significantly higher population of CD8 T cells that recognized immunodominant RSV peptides by day 8 post-infection in MLN (Fig. 6B). COX-2 inhibition resulted in a small, but not significant, increase in RSV-specific CD8 T cells in the lung of geriatric cotton rats.

### COX-2 and PGDS expression is not elevated in geriatric cotton rats

**Fig 7:**
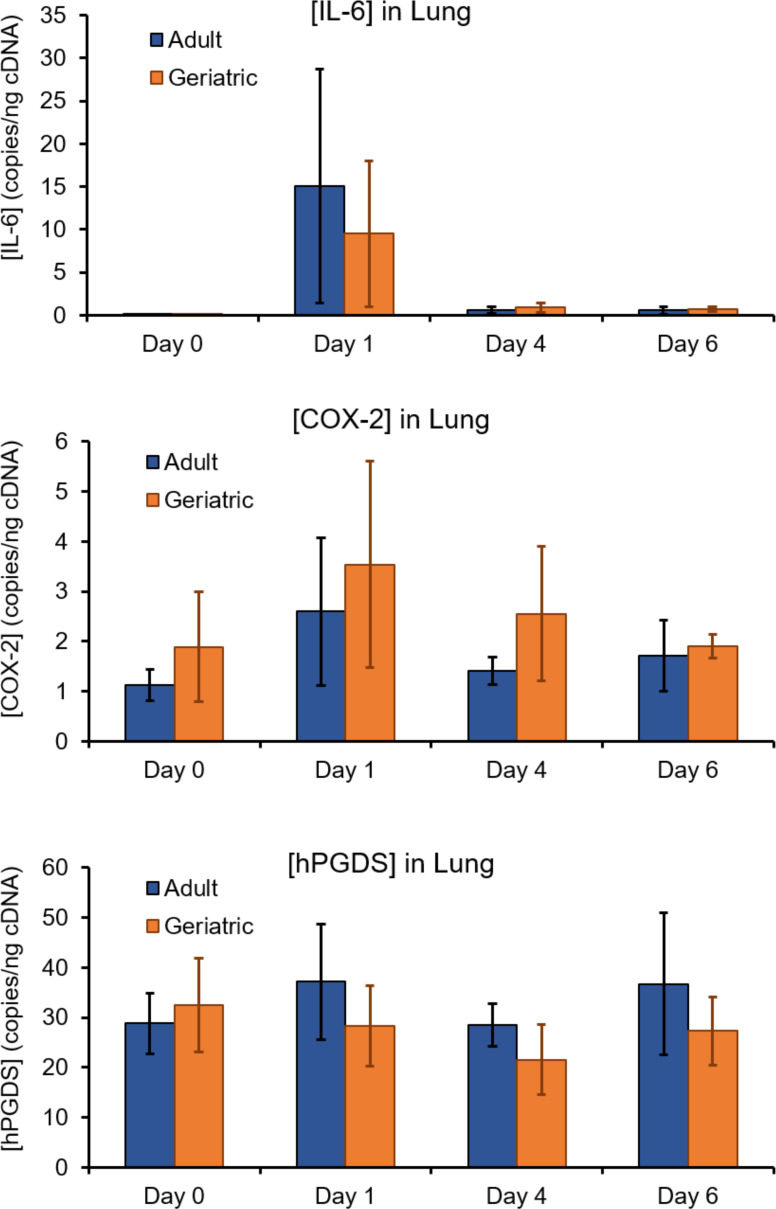
COX-2 and hPGDS genes are not upregulated in geriatric cotton rats compared with adults. The left lung lobe was collected from adult and geriatric cotton rats at the indicated time points post-RSV infection. Absolute quantification of IL-6, COX-2, and hPGDS copies was measured by ddPCR of 300 ng of cDNA for each sample. IL-6, a cytokine known to be upregulated early in RSV infection in cotton rats, displays an expected spike in expression at day 1 post-infection. Although an approximately 2-fold increase is seen at day 1 over day 0 for COX-2, neither COX-2 nor hPGDS differs significantly in expression throughout the course of infection compared with baseline (day 0). At baseline and all measured time points following infection, geriatric and adult cotton rats express similar concentrations of COX-2 and hPGDS mRNA. (*n* = 5 for all adult time points and geriatric day 6. *n* = 5 for geriatric days 0, 1, and 4.)

One mechanism that could result in elevated PGD2 production in geriatric cotton rats during RSV infection is increased expression of the genes for COX-2 or PGDS. Precedent for age-related increases in COX-2 gene expression has been shown in the heart of rats, although this was at the steady state and in the absence of an inflammatory condition (31). Hematopoietic PGDS (hPGDS), the primary isoform responsible for PGD2 generation in the lung, is upregulated in primary human airway epithelial cells from children following RSV infection (32). To determine whether these enzymes are upregulated transcriptionally in geriatric cotton rats, both over the course of RSV infection and at baseline, we performed digital droplet PCR on left lung lobes from adult and geriatric cotton rats at days 0, 1, 4, and 6 post-infection with RSV to obtain absolute quantification of gene transcripts. In addition to measuring COX-2 and hPGDS, we measured interleukin-6 (IL-6) as a known upregulated cytokine during RSV infection in cotton rats to serve as a positive control. Positive event counts were generated by ddPCR for all genes, indicating detectable expression. IL-6 expression is markedly increased at day 1 post-infection compared with baseline for both adult and geriatric cotton rats (Fig. 7), as previously reported (33). Average COX-2 expression increased approximately 2-fold from baseline to day 1 post-infection in both age groups, but this was not a statistically significant result. Variation in hPGDS expression was minimal throughout the course of infection. Both at baseline and during RSV infection, expression of COX-2 and hPGDS was similar in both age groups (Fig. 7).

## DISCUSSION

It is currently not understood how pathways involved in normal antiviral responses to RSV are affected by aging. Like humans, geriatric cotton rats exhibit a defective immune response to RSV characterized by delayed clearance. In this study, we characterized an inflammatory pathway that results in age-associated impairment of RSV clearance in the geriatric cotton rat model.

CD8+ T cells are important for the clearance of RSV infections (34, 35). Age-associated T-cell deficits are a well-described phenomenon, resulting in increased susceptibility to numerous infectious agents including respiratory viruses (36–39). There are numerous mechanisms contributing to impaired T-cell responses in the elderly, including thymic involution and a declining repertoire of naïve T cells (40, 41) as well as a rising proportion of T cells with an exhausted/senescent phenotype (42). A consequence of senescence of T cells and other leukocytes is an upregulation of proinflammatory cytokines including tumor necrosis factor and IL-6, which in turn may lead to more rapid aging of immune cells (39, 43, 44) and a concomitant rise in immunomodulatory regulatory T cells (45, 46). This observation has led researchers to hypothesize about the potential for anti-inflammatory therapies in elderly populations to combat an age-associated pro-inflammatory state (47). Anti-inflammatory intervention improved immune parameters in some experimental settings, including slowing the accumulation of telomere defects (48), improving memory T-cell responses to varicella zoster virus immunization (49), and enhancing antibody titers following influenza vaccination (50). We have previously shown that the non-steroidal anti-inflammatory drug (NSAID) ibuprofen improves RSV clearance in geriatric cotton rats (20), leading us to investigate whether cell-mediated immunity is impaired by an inflammatory pathway involving the COX enzymes.

We demonstrated that generation of a robust RSV-specific CD8+ T-cell response is delayed in geriatric cotton rats, correlating with their prolonged clearance kinetic. DCs play a critical role in the activation of cytotoxic T cells following RSV infection, providing MHC class-I-mediated antigen presentation, co-stimulation, and cytokines including type I IFNs and IL-12 to naive CD8+ T cells. Thus, we measured DC activation and migration in RSV-infected cotton rats. We found that CCR7 expression in lung DCs was decreased in the first 2 days of infection in geriatric cotton rats compared with adults. CCR7 is not only a useful marker of activation in DCs but also a key receptor for chemotaxis to regional lymph nodes mediated by CCL19 and CCL21. In consequence, we observed that DC influx into MLNs was decreased early in infection in geriatric cotton rats. In light of our previous finding that ibuprofen restores RSV clearance in geriatric cotton rats (20), we investigated PGD2 as a possible source of defective DC migration and CD8+ T-cell responses in our model. We found that specific agonism of the DP1 receptor in young cohorts recapitulates delayed clearance seen in geriatric cohorts. Inhibition of COX-2 or the more specific PGDS significantly improved DC and CD8+ T-cell responses and was sufficient to restore clearance kinetics in geriatric cotton rats. We did not find elevated expression of COX-2 or hPGDS in the lung of geriatric cotton rats compared with adults, either at baseline or throughout RSV infection. This suggests that these genes are not differentially upregulated in geriatric cotton rats compared with adults, although it remains possible that a specific cell population displays differential gene expression that was not captured by our analysis of whole lungs. Alternatively, the increase in PGD2 generation in RSV-infected geriatric cotton rats may be a consequence of post-transcriptional regulation resulting in increased COX-2 or PGDS activity. Our finding that DC migration and CD8+ T-cell responses are impaired in geriatric cotton rats does not preclude the presence of other age-associated immune deficits in this model. Our own data show that RSV-specific B cell responses and antibody titers are lower in geriatric cotton rats. These parameters were not improved by ibuprofen treatment, suggesting a separate mechanism for impaired humoral immunity (20).

Our findings align with those of several previous studies of CD8+ T-cell responses, DC migration, and the effect of PGD2 in mouse models with RSV and other respiratory viruses. Impaired CD8+ T-cell responses have been shown in aged RSV-infected BALB/c mice. Cytotoxic T-cell responses in aged mice were found to be reduced in number but not quality, with no differences in effector cytokine production between age groups (36). Impaired DC migration has been demonstrated in aged mice infected with RSV, influenza A, and SARS CoV-1 (24). That study also implicated an age-associated elevation in PGD2 as the mediator of this impaired DC migration during influenza and SARS CoV-1 infection; a later study showed that PGD2 was also detrimental in aged mice during SARS CoV-2 infection (29). A key difference is seen in the relative impact of DP1 and DP2 activity in RSV infection between young and old individuals. In primary airway epithelial cells from young children, DP1 agonism reduced RSV replication and promoted IFN-λ production (32). DP1 agonism in neonatal mice infected with pneumonia virus of mice, a relative of RSV, also resulted in improved antiviral immunity characterized by increased IFN-λ production and reduced viral load, whereas DP2 antagonism resulted in lower viral loads and lower markers of type 2 inflammation (32, 51). However, the heightened immune response in DP1 agonist-treated neonatal mice also resulted in the production of pathologic levels of TNF-α, IL-6, and IL-1β. These results in young mice and patient samples contrast with those seen in old individuals and animal models, highlighting the fact that increased susceptibility to severe RSV-associated disease in neonatal and elderly individuals is due to separate mechanisms, with immunopathology playing a more important role in infants (52–55).

Although studies in humans investigating the effects of COX inhibition during respiratory infections (via either NSAID or selective COX-2 inhibitor administration) are far outnumbered by *in vitro* and *in vivo* experiments, several analyses have been published. A matched prospective cohort study found no evidence that NSAID use in the 2 weeks leading up to hospitalization with SARS-CoV-2 resulted in poorer clinical outcomes (56). A meta-analysis of 40 comparative studies similarly concluded that there was no evidence of adverse outcomes resulting from NSAID use during SARS-CoV-2 infection (57). In a cohort study, no association was found between NSAID use and intensive care unit administration or mortality in influenza patients (58). Fewer human studies have directly examined the potential beneficial effect of NSAID or COX-2 inhibitors during respiratory infections. A randomized clinical trial found that indomethacin treatment in SARS-CoV 2 patients resulted in decreased rates of oxygen desaturation and more rapid symptom relief compared with those receiving other adjuvant therapies (59). A retrospective cohort study found that SARS-CoV 2 patients treated with indomethacin in concert with other adjuvant therapies within 72 hours of the onset of symptoms experienced a reduced duration of disease and lower risk of hospitalization, compared with those receiving delayed treatment (60). However, indomethacin exhibits direct antiviral against SARS-CoV-1 and related coronaviruses, meaning that the beneficial effects of indomethacin treatment of SARS-CoV-2 may not be due to anti-inflammatory effects (61). A phase 2 clinical trial for asapiprant (BGE-175), a DP1 inhibitor, in hospitalized COVID-19 patients was initiated in 2021 but is listed as Terminated, citing a drop in hospitalization rates that rendered the study no longer feasible (62).

For many infectious diseases, the elderly are not only more susceptible to severe clinical outcomes but also less responsive to immunization efforts (63). The elderly are an important target population for vaccination efforts, and mitigating impaired vaccine efficacy and secondary immunity in general is an important area of research. As with the age-related decline in many immune parameters, both immunosenescence phenotypes and inflammaging play a role in declining responsiveness to vaccination. Several mechanisms by which inflammaging leads to decreased secondary immune responses have been studied. Inflammaging-associated cytokines can increase the levels of inhibitory ligands on immune cells, such as elevated PDL-1 expression on antigen-presenting cells induced by TNF-α (64). In mice, TNF-α has also been shown to recruit Foxp3 + Treg cells and increase their immunosuppressive function (65). An increased number of Treg cells in the skin of the elderly both at baseline and upon antigen stimulation may contribute to decreased cutaneous immunity (66, 67). Inflammaging-associated cytokines also contribute to immunosenescence phenotypes such as decreased T-cell receptor signaling and monocyte phagocytosis (68, 69). There is increasing evidence that anti-inflammatory treatment may be beneficial in countering inflammaging and strengthening vaccine responses in the elderly (70–72). Monocyte inflammatory signatures are negatively correlated with influenza and yellow fever antibody titers following vaccination (73, 74). Increased expression of inflammatory genes prior to hepatitis B vaccination is predictive of a weaker response to the vaccine (75, 76). Increased monocyte COX-2 expression and prostaglandin E_2_ production inhibit immunity to varicella zoster virus antigen challenge in the elderly (77). Although these studies suggest that anti-inflammatory treatment may have beneficial effects on secondary immune responses, direct evidence in humans is lacking.

One limitation of our study is the fact that we model the delay in immune responses in geriatric cotton rats after primary RSV infection. This is in contrast to humans who encounter repeated RSV infections throughout their lifetime, and inhibition of immune responses in the elderly is an impairment of memory responses and not the generation of a primary immune response. Any differences in memory responses between adult and geriatric cotton rats are not assessed by our model. Further studies in humans should address whether mechanistic findings from the cotton rat (and for other respiratory infections the mouse) model can be applied to suboptimal secondary immune responses in aging humans.

In summary, our results show that RSV-specific CD8+ T-cell responses are delayed in geriatric cotton rats as a consequence of decreased DC activation and migration to MLNs. We attribute impaired DC activation to elevated PGD2 production elicited by RSV infection in geriatric cotton rats. Mimicking elevated PGD2 in adult animals through DP1 agonism resulted in delayed RSV clearance while inhibiting PGD2 production in geriatric animals restored clearance and improved DC activation and RSV-specific CD8+ T-cell responses. Future studies could consider identifying safe and effective targeted therapeutic interventions that inhibit PGDS activity or DP1 signaling for use in elderly individuals.

## Data Availability

No novel structural determinations were generated. All sequences used are available in GenBank under the accession numbers described. All reagents utilized in this study are commercially available, and any assistance with our described procedures will be provided upon request.

## References

[1] Hansen CL, Chaves SS, Demont C, Viboud C. 2022. Mortality associated with influenza and respiratory syncytial virus in the US, 1999-2018. JAMA Netw Open 5:e220527. doi:10.1001/jamanetworkopen.2022.0527 35226079 PMC8886548

[2] Matias G, Taylor R, Haguinet F, Schuck-Paim C, Lustig R, Shinde V. 2014. Estimates of mortality attributable to influenza and RSV in the United States during 1997-2009 by influenza type or subtype, age, cause of death, and risk status. Influenza Other Respir Viruses 8:507–515. doi:10.1111/irv.12258 24975705 PMC4181813

[3] Zheng Z, Warren JL, Shapiro ED, Pitzer VE, Weinberger DM. 2022. Estimated incidence of respiratory hospitalizations attributable to RSV infections across age and socioeconomic groups. Pneumonia (Nathan) 14:6. doi:10.1186/s41479-022-00098-x 36280891 PMC9592130

[4] McLaughlin JM, Khan F, Begier E, Swerdlow DL, Jodar L, Falsey AR. 2022. Rates of medically attended RSV among US adults: a systematic review and meta-analysis. Open Forum Infect Dis 9:fac300. doi:10.1093/ofid/ofac300 PMC9301578 35873302

[5] Fulton RB, Varga SM. 2009. Effects of aging on the adaptive immune response to respiratory virus infections. Aging health 5:775. doi:10.2217/ahe.09.69 20174457 PMC2822389

[6] Busse PJ, Mathur SK. 2010. Age-related changes in immune function: effect on airway inflammation. J Allergy Clin Immunol 126:690–699; doi:10.1016/j.jaci.2010.08.011 20920759 PMC3297963

[7] Green MG, Huey D, Niewiesk S. 2013. The cotton rat (Sigmodon hispidus) as an animal model for respiratory tract infections with human pathogens. Lab Anim 42:170–176. doi:10.1038/laban.188 23604159

[8] Boukhvalova MS, Prince GA, Blanco JCG. 2009. The cotton rat model of respiratory viral infections. Biologicals 37:152–159. doi:10.1016/j.biologicals.2009.02.017 19394861 PMC2882635

[9] Prince GA, Mathews A, Curtis SJ, Porter DD. 2000. Treatment of respiratory syncytial virus bronchiolitis and pneumonia in a cotton rat model with systemically administered monoclonal antibody (palivizumab) and glucocorticosteroid. J infect dis 182:1326–1330. doi:10.1086/315894

[10] Boukhvalova MS, Yim KC, Blanco J. 2018. Cotton rat model for testing vaccines and antivirals against respiratory syncytial virus. Antivir Chem Chemother 26:2040206618770518. doi:10.1177/2040206618770518 29768937 PMC5987903

[11] Zhang L, Peeples ME, Boucher RC, Collins PL, Pickles RJ. 2002. Respiratory syncytial virus infection of human airway epithelial cells is polarized, specific to ciliated cells, and without obvious cytopathology. J Virol 76:5654–5666. doi:10.1128/jvi.76.11.5654-5666.2002 11991994 PMC137037

[12] Grieves JL, Yin Z, Durbin RK, Durbin JE. 2015. Acute and chronic airway disease after human respiratory syncytial virus infection in cotton rats (Sigmodon hispidus). Comp Med 65:315–326. 26310461 PMC4549677

[13] Prince GA, Horswood RL, Camargo E, Koenig D, Chanock RM. 1983. Mechanisms of immunity to respiratory syncytial virus in cotton rats. Infect Immun 42:81–87. doi:10.1128/iai.42.1.81-87.1983 6352505 PMC264527

[14] Byrd LG, Prince GA. 1997. Animal models of respiratory syncytial virus infection. Clin Infect Dis 25:1363–1368. doi:10.1086/516152 9431379

[15] Wethington D, Harder O, Uppulury K, Stewart WCL, Chen P, King T, Reynolds SD, Perelson AS, Peeples ME, Niewiesk S, Das J. 2019. Mathematical modelling identifies the role of adaptive immunity as a key controller of respiratory syncytial virus in cotton rats. J R Soc Interface 16:20190389. doi:10.1098/rsif.2019.0389 31771450 PMC6893489

[16] Yamaji Y, Nakayama T. 2014. Recombinant measles viruses expressing respiratory syncytial virus proteins induced virus-specific CTL responses in cotton rats. Vaccine (Auckl) 32:4529–4536. doi:10.1016/j.vaccine.2014.06.024 24951869

[17] Yamaji Y, Sawada A, Yasui Y, Ito T, Nakayama T. 2019. Simultaneous administration of recombinant measles viruses expressing respiratory syncytial virus fusion (F) and nucleo (N) proteins induced humoral and cellular immune responses in cotton rats. Vaccines (Basel) 7:27. doi:10.3390/vaccines7010027 30836661 PMC6466305

[18] Zhan X, Slobod KS, Jones BG, Sealy RE, Takimoto T, Boyd K, Surman S, Russell CJ, Portner A, Hurwitz JL. 2015. Sendai virus recombinant vaccine expressing a secreted, unconstrained respiratory syncytial virus fusion protein protects against RSV in cotton rats. Int Immunol 27:229–236. doi:10.1093/intimm/dxu107 25477211 PMC4406265

[19] Curtis SJ, Ottolini MG, Porter DD, Prince GA. 2002. Age-dependent replication of respiratory syncytial virus in the cotton rat. Exp Biol Med (Maywood) 227:799–802. doi:10.1177/153537020222700912 12324660

[20] Harder OE, Martinez M, Niewiesk S. 2021. Nonsteroidal anti-inflammatory drugs restore immune function to respiratory syncytial virus in geriatric cotton rats (Sigmodon hispidus). Virology (Auckl) 563:28–37. doi:10.1016/j.virol.2021.08.006 34411809

[21] Boukhvalova MS, Yim KC, Kuhn KH, Hemming JP, Prince GA, Porter DD, Blanco JCG. 2007. Age-related differences in pulmonary cytokine response to respiratory syncytial virus infection: modulation by anti-inflammatory and antiviral treatment. J Infect Dis 195:511–518. doi:10.1086/510628 17230410

[22] Faith RE, Montgomery CA, Durfee WJ, Aguilar-Cordova E, Wyde PR. 1997. The cotton rat in biomedical research. Lab Anim Sci 47:337–345. 9306305

[23] Guichelaar T, van Erp EA, Hoeboer J, Smits NAM, van Els CACM, Pieren DKJ, Luytjes W. 2018. Diversity of aging of the immune system classified in the cotton rat (Sigmodon hispidus) model of human infectious diseases. Dev Comp Immunol 82:39–48. doi:10.1016/j.dci.2017.12.026 29305168

[24] Zhao J, Zhao J, Legge K, Perlman S. 2011. Age-related increases in PGD(2) expression impair respiratory DC migration, resulting in diminished T cell responses upon respiratory virus infection in mice. J Clin Invest 121:4921–4930. doi:10.1172/JCI59777 22105170 PMC3226008

[25] Hammad H, de Heer HJ, Soullie T, Hoogsteden HC, Trottein F, Lambrecht BN. 2003. Prostaglandin D2 inhibits airway dendritic cell migration and function in steady state conditions by selective activation of the D prostanoid receptor 1. J Immunol 171:3936–3940. doi:10.4049/jimmunol.171.8.3936 14530310

[26] Angeli V, Faveeuw C, Roye O, Fontaine J, Teissier E, Capron A, Wolowczuk I, Capron M, Trottein F. 2001. Role of the parasite-derived prostaglandin D2 in the inhibition of epidermal Langerhans cell migration during schistosomiasis infection. J Exp Med 193:1135–1147. doi:10.1084/jem.193.10.1135 11369785 PMC2193325

[27] Gosset P, Bureau F, Angeli V, Pichavant M, Faveeuw C, Tonnel A-B, Trottein F. 2003. Prostaglandin D2 affects the maturation of human monocyte-derived dendritic cells: consequence on the polarization of naive Th cells. J Immunol 170:4943–4952. doi:10.4049/jimmunol.170.10.4943 12734337

[28] Reed LJ, Muench H. 1938. A simple method of estimating fifty per cent endpoints. Am J Epidemiol 27:493–497. doi:10.1093/oxfordjournals.aje.a118408

[29] Wong L-YR, Zheng J, Wilhelmsen K, Li K, Ortiz ME, Schnicker NJ, Thurman A, Pezzulo AA, Szachowicz PJ, Li P, Pan R, Klumpp K, Aswad F, Rebo J, Narumiya S, Murakami M, Zuniga S, Sola I, Enjuanes L, Meyerholz DK, Fortney K, McCray PB Jr, Perlman S. 2022. Eicosanoid signalling blockade protects middle-aged mice from severe COVID-19. Nature New Biol 605:146–151. doi:10.1038/s41586-022-04630-3 PMC9783543 35314834

[30] Angeli V, Staumont D, Charbonnier A-S, Hammad H, Gosset P, Pichavant M, Lambrecht BN, Capron M, Dombrowicz D, Trottein F. 2004. Activation of the D prostanoid receptor 1 regulates immune and skin allergic responses. J Immunol 172:3822–3829. doi:10.4049/jimmunol.172.6.3822 15004188

[31] Kim JW, Baek BS, Kim YK, Herlihy JT, Ikeno Y, Yu BP, Chung HY. 2001. Gene expression of cyclooxygenase in the aging heart. J Gerontol A Biol Sci Med Sci 56:B350–5. doi:10.1093/gerona/56.8.b350 11487593

[32] Werder RB, Lynch JP, Simpson JC, Zhang V, Hodge NH, Poh M, Forbes-Blom E, Kulis C, Smythe ML, Upham JW, Spann K, Everard ML, Phipps S. 2018. PGD2/DP2 receptor activation promotes severe viral bronchiolitis by suppressing IFN-**λ** production. Sci Transl Med 10:eaao0052. doi:10.1126/scitranslmed.aao0052 29743346

[33] Blanco JCG, Richardson JY, Darnell MER, Rowzee A, Pletneva L, Porter DD, Prince GA. 2002. Cytokine and chemokine gene expression after primary and secondary respiratory syncytial virus infection in cotton rats. J Infect Dis 185:1780–1785. doi:10.1086/340823 12085325

[34] Morabito KM, Erez N, Graham BS, Ruckwardt TJ. 2016. Phenotype and hierarchy of two transgenic T cell lines targeting the respiratory syncytial virus KdM282-90 epitope is transfer dose-dependent. PLoS ONE 11:e0146781. doi:10.1371/journal.pone.0146781 26752171 PMC4708989

[35] Graham BS, Bunton LA, Wright PF, Karzon DT. 1991. Role of T lymphocyte subsets in the pathogenesis of primary infection and rechallenge with respiratory syncytial virus in mice. J Clin Invest 88:1026–1033. doi:10.1172/JCI115362 1909350 PMC295511

[36] Fulton RB, Weiss KA, Pewe LL, Harty JT, Varga SM. 2013. Aged mice exhibit a severely diminished CD8 T cell response following respiratory syncytial virus infection. J Virol 87:12694–12700. doi:10.1128/JVI.02282-12 24049171 PMC3838124

[37] Bevilacqua A, Ho P-C, Franco F. 2023. Metabolic reprogramming in inflammaging and aging in T cells. Life Met 2:load028. doi:10.1093/lifemeta/load028 PMC11749375 39872627

[38] Parks OB, Eddens T, Sojati J, Lan J, Zhang Y, Oury TD, Ramsey M, Erickson JJ, Byersdorfer CA, Williams JV. 2023. Terminally exhausted CD8+ T cells contribute to age-dependent severity of respiratory virus infection. Immun Ageing 20:40. doi:10.1186/s12979-023-00365-5 37528458 PMC10391960

[39] Mittelbrunn M, Kroemer G. 2021. Hallmarks of T cell aging. Nat Immunol 22:687–698. doi:10.1038/s41590-021-00927-z 33986548

[40] Lynch HE, Goldberg GL, Chidgey A, Van den Brink MRM, Boyd R, Sempowski GD. 2009. Thymic involution and immune reconstitution. Trends Immunol 30:366–373. doi:10.1016/j.it.2009.04.003 19540807 PMC2750859

[41] Yager EJ, Ahmed M, Lanzer K, Randall TD, Woodland DL, Blackman MA. 2008. Age-associated decline in T cell repertoire diversity leads to holes in the repertoire and impaired immunity to influenza virus. J Exp Med 205:711–723. doi:10.1084/jem.20071140 18332179 PMC2275391

[42] Rodriguez IJ, Lalinde Ruiz N, Llano León M, Martínez Enríquez L, Montilla Velásquez MDP, Ortiz Aguirre JP, Rodríguez Bohórquez OM, Velandia Vargas EA, Hernández ED, Parra López CA. 2020. Immunosenescence study of T cells: a systematic review. Front Immunol 11:604591. doi:10.3389/fimmu.2020.604591 33519813 PMC7843425

[43] Pera A, Campos C, López N, Hassouneh F, Alonso C, Tarazona R, Solana R. 2015. Immunosenescence: implications for response to infection and vaccination in older people. Maturitas 82:50–55. doi:10.1016/j.maturitas.2015.05.004 26044074

[44] Desdín-Micó G, Soto-Heredero G, Aranda JF, Oller J, Carrasco E, Gabandé-Rodríguez E, Blanco EM, Alfranca A, Cussó L, Desco M, Ibañez B, Gortazar AR, Fernández-Marcos P, Navarro MN, Hernaez B, Alcamí A, Baixauli F, Mittelbrunn M. 2020. T cells with dysfunctional mitochondria induce multimorbidity and premature senescence. Science 368:1371–1376. doi:10.1126/science.aax0860 32439659 PMC7616968

[45] Sharma S, Dominguez AL, Lustgarten J. 2006. High accumulation of T regulatory cells prevents the activation of immune responses in aged animals. J Immunol 177:8348–8355. doi:10.4049/jimmunol.177.12.8348 17142731

[46] Nishioka T, Shimizu J, Iida R, Yamazaki S, Sakaguchi S. 2006. CD4+CD25+Foxp3+ T cells and CD4+CD25-Foxp3+ T cells in aged mice. J Immunol 176:6586–6593. doi:10.4049/jimmunol.176.11.6586 16709816

[47] Akbar AN, Gilroy DW. 2020. Aging immunity may exacerbate COVID-19. Science 369:256–257. doi:10.1126/science.abb0762 32675364

[48] Jurk D, Wilson C, Passos JF, Oakley F, Correia-Melo C, Greaves L, Saretzki G, Fox C, Lawless C, Anderson R, Hewitt G, Pender SL, Fullard N, Nelson G, Mann J, van de Sluis B, Mann DA, von Zglinicki T. 2014. Chronic inflammation induces telomere dysfunction and accelerates ageing in mice. Nat Commun 2:4172. doi:10.1038/ncomms5172 24960204 PMC4090717

[49] Vukmanovic-Stejic M, Chambers ES, Suárez-Fariñas M, Sandhu D, Fuentes-Duculan J, Patel N, Agius E, Lacy KE, Turner CT, Larbi A, Birault V, Noursadeghi M, Mabbott NA, Rustin MHA, Krueger JG, Akbar AN. 2018. Enhancement of cutaneous immunity during aging by blocking p38 mitogen-activated protein (MAP) kinase-induced inflammation. J Allergy Clin Immunol 142:844–856. doi:10.1016/j.jaci.2017.10.032 29155150 PMC6127037

[50] Mannick JB, Morris M, Hockey H-UP, Roma G, Beibel M, Kulmatycki K, Watkins M, Shavlakadze T, Zhou W, Quinn D, Glass DJ, Klickstein LB. 2018. TORC1 inhibition enhances immune function and reduces infections in the elderly. Sci Transl Med 10:eaaq1564. doi:10.1126/scitranslmed.aaq1564 29997249

[51] Ullah MA, Rittchen S, Li J, Hasnain SZ, Phipps S. 2021. DP1 prostanoid receptor activation increases the severity of an acute lower respiratory viral infection in mice via TNF-α-induced immunopathology. Mucosal Immunol 14:963–972. doi:10.1038/s41385-021-00405-7 33879829 PMC8057290

[52] McNamara PS, Smyth RL. 2002. The pathogenesis of respiratory syncytial virus disease in childhood. Br Med Bull 61:13–28. doi:10.1093/bmb/61.1.13 11997296

[53] Christiaansen AF, Syed MA, Ten Eyck PP, Hartwig SM, Durairaj L, Kamath SS, Varga SM. 2016. Altered Treg and cytokine responses in RSV-infected infants. Pediatr Res 80:702–709. doi:10.1038/pr.2016.130 27486703 PMC6215710

[54] Bergeron HC, Tripp RA. 2021. Immunopathology of RSV: an updated review. Viruses 13:2478. doi:10.3390/v13122478 34960746 PMC8703574

[55] Turi KN, Shankar J, Anderson LJ, Rajan D, Gaston K, Gebretsadik T, Das SR, Stone C, Larkin EK, Rosas-Salazar C, Brunwasser SM, Moore ML, Peebles RS Jr, Hartert TV. 2018. Infant viral respiratory infection nasal immune-response patterns and their association with subsequent childhood recurrent wheeze. Am J Respir Crit Care Med 198:1064–1073. doi:10.1164/rccm.201711-2348OC 29733679 PMC6221572

[56] Drake TM, Fairfield CJ, Pius R, Knight SR, Norman L, Girvan M, Hardwick HE, Docherty AB, Thwaites RS, Openshaw PJM, Baillie JK, Harrison EM, Semple MG, ISARIC4C Investigators. 2021. Non-steroidal anti-inflammatory drug use and outcomes of COVID-19 in the ISARIC Clinical Characterisation Protocol UK cohort: a matched, prospective cohort study. Lancet Rheumatol 3:e498–e506. doi:10.1016/S2665-9913(21)00104-1 33997800 PMC8104907

[57] Zhou Q, Zhao S, Gan L, Wang Z, Peng S, Li Q, Liu H, Liu X, Wang Z, Shi Q, Estill J, Luo Z, Wang X, Liu E, Chen Y. 2022. Use of non-steroidal anti-inflammatory drugs and adverse outcomes during the COVID-19 pandemic: a systematic review and meta-analysis. EClinMed 46:101373. doi:10.1016/j.eclinm.2022.101373 PMC8989274 35434582

[58] Lund LC, Reilev M, Hallas J, Kristensen KB, Thomsen RW, Christiansen CF, Sørensen HT, Johansen NB, Brun NC, Voldstedlund M, Støvring H, Thomsen MK, Christensen S, Pottegård A. 2020. Association of nonsteroidal anti-inflammatory drug use and adverse outcomes among patients hospitalized with influenza. JAMA Netw Open 3:e2013880. doi:10.1001/jamanetworkopen.2020.13880 32609352 PMC7330719

[59] Ravichandran R, Mohan SK, Sukumaran SK, Kamaraj D, Daivasuga SS, Ravi SOAS, Vijayaraghavalu S, Kumar RK. 2022. An open label randomized clinical trial of Indomethacin for mild and moderate hospitalised Covid-19 patients. Sci Rep 12:6413. doi:10.1038/s41598-022-10370-1 35440611 PMC9016692

[60] Fazio S, Bellavite P, Zanolin E, McCullough PA, Pandolfi S, Affuso F. 2021. Retrospective study of outcomes and hospitalization rates of patients in Italy with a confirmed diagnosis of early COVID-19 and treated at home within 3 days or after 3 days of symptom onset with prescribed and non-prescribed treatments between November 2020 and August 2021. Med Sci Monit 27:e935379. doi:10.12659/MSM.935379 34966165 PMC8725339

[61] Amici C, Di Caro A, Ciucci A, Chiappa L, Castilletti C, Martella V, Decaro N, Buonavoglia C, Capobianchi MR, Santoro MG. 2006. Indomethacin has a potent antiviral activity against SARS coronavirus. Antivir Ther 11:1021–1030. 17302372

[62] Wilkerson R. 2023. Study to evaluate the safety, tolerability, and efficacy of BGE-175 in hospitalized adults with coronavirus disease 2019 (COVID-19) that are not in respiratory failure. ClinTrialsgov Avail via. Available from: https://clinicaltrials.gov/study/NCT04705597. Retrieved 28 May 2024.

[63] Aspinall R, Del Giudice G, Effros RB, Grubeck-Loebenstein B, Sambhara S. 2007. Challenges for vaccination in the elderly. Immun Ageing 4:9. doi:10.1186/1742-4933-4-9 18072962 PMC2235886

[64] Lim S-O, Li C-W, Xia W, Cha J-H, Chan L-C, Wu Y, Chang S-S, Lin W-C, Hsu J-M, Hsu Y-H, Kim T, Chang W-C, Hsu JL, Yamaguchi H, Ding Q, Wang Y, Yang Y, Chen C-H, Sahin AA, Yu D, Hortobagyi GN, Hung M-C. 2016. Deubiquitination and stabilization of PD-L1 by CSN5. Cancer Cell 30:925–939. doi:10.1016/j.ccell.2016.10.010 27866850 PMC5171205

[65] Chen X, Bäumel M, Männel DN, Howard OMZ, Oppenheim JJ. 2007. Interaction of TNF with TNF receptor type 2 promotes expansion and function of mouse CD4+CD25+ T regulatory cells. J Immunol 179:154–161. doi:10.4049/jimmunol.179.1.154 17579033

[66] Vukmanovic-Stejic M, Sandhu D, Sobande TO, Agius E, Lacy KE, Riddell N, Montez S, Dintwe OB, Scriba TJ, Breuer J, Nikolich-Zugich J, Ogg G, Rustin MHA, Akbar AN. 2013. Varicella zoster-specific CD4+Foxp3+ T cells accumulate after cutaneous antigen challenge in humans. J Immunol 190:977–986. doi:10.4049/jimmunol.1201331 23284056 PMC3552094

[67] Vukmanovic-Stejic M, Agius E, Booth N, Dunne PJ, Lacy KE, Reed JR, Sobande TO, Kissane S, Salmon M, Rustin MH, Akbar AN. 2008. The kinetics of CD4+Foxp3+ T cell accumulation during a human cutaneous antigen-specific memory response in vivo. J Clin Invest 118:3639–3650. doi:10.1172/JCI35834 18924611 PMC2556297

[68] Cope AP, Liblau RS, Yang XD, Congia M, Laudanna C, Schreiber RD, Probert L, Kollias G, McDevitt HO. 1997. Chronic tumor necrosis factor alters T cell responses by attenuating T cell receptor signaling. J Exp Med 185:1573–1584. doi:10.1084/jem.185.9.1573 9151895 PMC2196294

[69] Puchta A, Naidoo A, Verschoor CP, Loukov D, Thevaranjan N, Mandur TS, Nguyen P-S, Jordana M, Loeb M, Xing Z, Kobzik L, Larché MJ, Bowdish DME. 2016. TNF drives monocyte dysfunction with age and results in impaired anti-pneumococcal immunity. PLoS Pathog 12:e1005368. doi:10.1371/journal.ppat.1005368 26766566 PMC4713203

[70] Chambers ES, Akbar AN. 2020. Can blocking inflammation enhance immunity during aging? J Allergy Clin Immunol 145:1323–1331. doi:10.1016/j.jaci.2020.03.016 32386656

[71] Wagner A, Weinberger B. 2020. Vaccines to prevent infectious diseases in the older population: immunological challenges and future perspectives. Front Immunol 11:717. doi:10.3389/fimmu.2020.00717 32391017 PMC7190794

[72] Pereira B, Xu X-N, Akbar AN. 2020. Targeting inflammation and immunosenescence to improve vaccine responses in the elderly. Front Immunol 11:583019. doi:10.3389/fimmu.2020.583019 33178213 PMC7592394

[73] Muyanja E, Ssemaganda A, Ngauv P, Cubas R, Perrin H, Srinivasan D, Canderan G, Lawson B, Kopycinski J, Graham AS, et al.. 2014. Immune activation alters cellular and humoral responses to yellow fever 17D vaccine. J Clin Invest 124:3147–3158. doi:10.1172/JCI75429 24911151 PMC4071376

[74] Nakaya HI, Hagan T, Duraisingham SS, Lee EK, Kwissa M, Rouphael N, Frasca D, Gersten M, Mehta AK, Gaujoux R, Li G-M, Gupta S, Ahmed R, Mulligan MJ, Shen-Orr S, Blomberg BB, Subramaniam S, Pulendran B. 2015. Systems analysis of immunity to influenza vaccination across multiple years and in diverse populations reveals shared molecular signatures. Immunity 43:1186–1198. doi:10.1016/j.immuni.2015.11.012 26682988 PMC4859820

[75] Fourati S, Cristescu R, Loboda A, Talla A, Filali A, Railkar R, Schaeffer AK, Favre D, Gagnon D, Peretz Y, Wang I-M, Beals CR, Casimiro DR, Carayannopoulos LN, Sékaly R-P. 2016. Pre-vaccination inflammation and B-cell signalling predict age-related hyporesponse to hepatitis B vaccination. Nat Commun 7:10369. doi:10.1038/ncomms10369 26742691 PMC4729923

[76] Bartholomeus E, De Neuter N, Meysman P, Suls A, Keersmaekers N, Elias G, Jansens H, Hens N, Smits E, Van Tendeloo V, Beutels P, Van Damme P, Ogunjimi B, Laukens K, Mortier G. 2018. Transcriptome profiling in blood before and after hepatitis B vaccination shows significant differences in gene expression between responders and non-responders. Vaccine (Auckl) 36:6282–6289. doi:10.1016/j.vaccine.2018.09.001 30205979

[77] Chambers ES, Vukmanovic-Stejic M, Shih BB, Trahair H, Subramanian P, Devine OP, Glanville J, Gilroy D, Rustin MHA, Freeman TC, Mabbott NA, Akbar AN. 2021. Recruitment of inflammatory monocytes by senescent fibroblasts inhibits antigen-specific tissue immunity during human aging. Nat Aging 1:101–113. doi:10.1038/s43587-020-00010-6 37118005

